# Metabolic reprogramming in efferocytosis

**DOI:** 10.3389/fcell.2025.1677028

**Published:** 2025-09-10

**Authors:** Qing Yan, Kuo Li, Lu Chen, Aowei Wang, Yingying Xi, Hui Xiao, Lei Yuan

**Affiliations:** ^1^ College of Life Sciences, Shaanxi Normal University, Xi’an, China; ^2^ Department of Human Anatomy and Histoembryology, School of Basic Medical Science, Ningxia Medical University, Yinchuan, China

**Keywords:** efferocytosis, apoptotic cell clearance, metabolic reprogramming, macrophages, glycolysis, fatty acid oxidation

## Abstract

Efferocytosis refers to the process by which phagocytes specifically identify and eliminate apoptotic cells. This process is essential for both maintaining tissue homeostasis and suppressing inflammatory responses, as well as facilitating tissue repair. When phagocytes internalize apoptotic cells, which act as “nutrient packages,” they undergo significant metabolic reprogramming. This reprogramming not only supplies energy and biosynthetic precursors necessary for engulfment but also critically influences the functional phenotype of phagocytes through complex molecular networks. These networks ultimately determine whether phagocytes adopt an anti-inflammatory resolution or a pathological pro-inflammatory state. This article offers a comprehensive analysis of the molecular regulatory mechanisms that underpin metabolic reprogramming during efferocytosis, aiming to elucidate the intricate regulatory networks formed by the interaction of metabolites as signaling molecules and classical signaling pathways. We examine how the three primary metabolic pathways—glucose, lipid, and amino acid metabolisms—are regulated by signals from efferocytosis and, in turn, modulate phagocyte function. A deeper understanding of the interplay between metabolic reprogramming and efferocytosis will provide a theoretical foundation and novel targets for treating diseases associated with impaired clearance of apoptotic cells.

## 1 Introduction

In multicellular organisms, the daily elimination of billions of cells through programmed cell death, predominantly apoptosis, is crucial for maintaining tissue homeostasis, aiding tissue repair, and ensuring normal development. This process of the clearance of apoptotic cells is known as “efferocytosis” (derived from Greek, meaning “to carry away the corpse”) (Boada-Romero et al., 2020; Moon et al., 2023; Qian et al., 2024). Successful efferocytosis not only prevents the secondary necrosis of apoptotic cells and the subsequent release of intracellular contents that can incite inflammation, but it also prompts phagocytes to secrete anti-inflammatory factors, such as IL-10 and TGF-β, along with growth factors that support tissue repair. This dual action aids in resolving inflammation and restoring tissue function (Yin and Heit, 2021; Rodríguez-Morales and Franklin, 2023). Therefore, impaired efferocytosis is closely linked to the onset and progression of various diseases, including atherosclerosis (Adkar and Leeper, 2024), systemic lupus erythematosus (SLE) (Li et al., 2024), chronic obstructive pulmonary disease (COPD) (Dutta et al., 2022), and neurodegenerative disorders (Zhang et al., 2022). Efferocytosis is carried out by both professional phagocytes, such as macrophages and dendritic cells, and non-professional phagocytes, like epithelial cells. This process involves a series of well-coordinated steps: apoptotic cells release “find-me” signals, including ATP and LPC, to draw phagocytes to their location. They also display “eat-me” signals, such as phosphatidylserine (PS), on their surface, which are identified by specific receptors on phagocytes, such as the TAM family receptors MerTK and Axl, as well as Tim-4 and BAI1 (Xiao et al., 2024). Following receptor engagement, downstream GTPases, such as Rac1, and adaptor proteins, including ELMO1/DOCK180, are activated, leading to cytoskeletal rearrangement. This process culminates in the engulfment of the apoptotic cell by the phagocyte, where it is subsequently degraded and processed within phagosomes (Katoh and Negishi, 2003; Grimsley et al., 2004; Wang et al., 2014).

Phagocytes face a considerable metabolic challenge when engulfing apoptotic cells. This process involves significant cytoskeletal rearrangement, membrane extension, and phagosome maturation, all of which are energy-intensive activities. Apoptotic cells are rich in biological macromolecules, including proteins, lipids, and nucleic acids. Once engulfed, these macromolecules are broken down in the phagolysosome into smaller nutrient molecules such as amino acids, fatty acids, cholesterol, and nucleosides (Medina et al., 2020; Schilperoort et al., 2023b). The entry of these nutrients into the cytoplasm triggers a substantial reprogramming of the phagocyte’s metabolic network, a phenomenon known as “metabolic reprogramming.” Initially, this reprogramming was considered a passive reaction to the influx of nutrients. However, pioneering research over the last decade has revealed that metabolic reprogramming is a pivotal factor in regulating efferocytosis. Metabolites like acetyl-CoA and kynurenine are intricately connected with classical immune signaling pathways, collectively shaping the efficiency and immunological outcomes of efferocytosis (Nong et al., 2023; Liu et al., 2024). This article will delve into the molecular mechanisms underlying this regulatory network, highlighting the essential role of metabolism in efferocytosis.

### 1.1 Efferocytosis

As an irreversible process, apoptosis is considered to be relatively conservative in evolution. Apoptosis cells recruit phagocytes through Find me signals such as signal molecules and chemokines, namely, nucleotides (ATP and UTP) (Elliott et al., 2009), membrane lipids such as phosphatidylserine (PS), sphingosine -1- phosphate (S1P) and lysophosphatidylcholine (LPC) (Peter et al., 2012). Phagocytes are recruited to the vicinity of apoptotic cells by Find me signal, and the apoptotic cells are accurately located by recognizing the Eat me signal released by apoptotic cells, and then specifically bind to the relevant receptors of phagocytes, so as to carry out phagocytosis. Eat me signals include Phosphatidylserine (PS) exposure (Zhang et al., 2012), changes in cell surface charges and glycosylation patterns (Khalaji et al., 2023), cell adhesion molecule 3(ICAM-3) (Starling et al., 1995; Kristóf et al., 2013), oxidized low-density lipoprotein (Zhang et al., 2017; Di and Maiseyeu, 2021) and endoplasmic reticulum. In addition, the expression of Don´t eat me signal serves as an additional regulatory mechanism to prevent improper clearance of living cells (Khalaji et al., 2023). Phagocytes recognize and combine with apoptotic cells through phagocytic receptors, that is, Eat me signals are transmitted into phagocytes, which leads to actin polymerization, cytoskeleton rearrangement, and phagocytes extend pseudopodes along the surface of apoptotic cells, which gradually extend to wrap apoptotic cells, and finally fuse to form “phagocytes” (Zhou and Yu, 2008). There are two main signal pathways in this phagocytosis process, which are described as MEGF10/GULP1 and RKII/Dock180/ELMO1 pathways in mice (Park et al., 2007; Tosello-Trampont et al., 2007; Zheng et al., 2017). After being phagocytized by phagocytes, apoptotic cells are degraded in phagocytes, and the new phagocytes fuse with early endosomes and late endosomes in turn, and the latter combine with lysosomes to form phagolysosomes, that is, phagolysosomes mature (Shklover et al., 2015; Hilu-Dadia et al., 2018). Rab GTPases proteins are needed for phagocyte maturation and subsequent acidification (Guo et al., 2010). RAB5 is located on the surface of early endosomes and new phagocytes, while RAB7 is specifically located on late endosomes, lysosomes and phagocytes. Under the relay action of Rab5 and Rab7, the structure of phagocyte and lysosome fused to form phagolysosome (Kinchen and Ravichandran, 2008). When phagocytosis lysosomes are formed, various membrane proteins, such as lysosomal Lys/Arg transporter LAAT-1, LMP 1, type II DNAse (NUC-1) and cathepsin L (CPL-1), are recruited, thus destroying and degrading apoptotic cells (Liu et al., 2012; Xu et al., 2014). In this process, lipids, nucleic acids, amino acids and other metabolites will be produced.

## 2 Lipid metabolism and efferocytosis

### 2.1 Lipid signaling molecules derived from apoptotic cells

**FIGURE 1:**
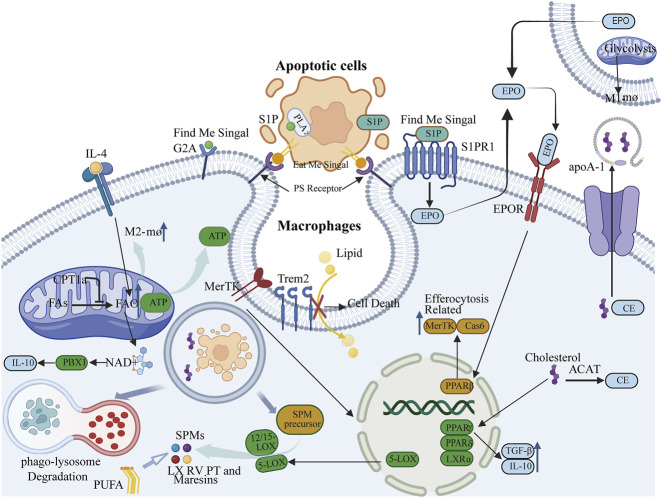
Lipid mediators such as lysophosphatidic acid (LPA), lysophosphatidylcholine (LPC), and sphingosine-1-phosphate (S1P) function as “find me” signals, whereas phosphatidylserine (PS) serves as an “eat me” signal, facilitating signal communication pathways essential for the initiation and progression of efferocytosis. S1P is also capable of activating the erythropoietin (EPO) signaling pathway, thereby influencing the peroxisome proliferator-activated receptor gamma (PPARγ) to upregulate Mer tyrosine kinase (MerTK) and caspase-6 (Cas6), which are molecules associated with efferocytosis. 2. Fatty acid oxidation (FAO) mediated by carnitine palmitoyltransferase 1a (CPT1a) provides energy necessary for the formation of efferocytic phagosomes and cytoskeletal rearrangement, and is critical for the activation of interleukin-4 (IL-4)-mediated M2 macrophages. 3. Macrophages sustain effective localization of PS receptors by storing or exporting cholesterol or cholesteryl esters derived from apoptotic cells. Concurrently, cholesterol can activate nuclear cholesterol receptors liver X receptor alpha (LXRα), PPARγ, and PPAR delta (PPARδ) to upregulate the expression of anti-inflammatory factors interleukin-10 (IL-10) and transforming growth factor beta (TGFβ), as well as the phagocytic receptor MerTK, thereby maintaining the stability of efferocytosis. 4. Phagocytes can absorb lipids through their surface lipid sensor, triggering receptor expressed on myeloid cells 2 (Trem2), and influence efferocytosis by regulating lipid metabolism. 5. Efferocytosis can modulate the production of the anti-inflammatory factor IL-10 and, through activation of the MerTK receptor, stimulate 5-lipoxygenase (5-LOX). In conjunction with 12/15-lipoxygenase (12/15-LOX), this catalyzes the production of specialized pro-resolving mediators (SPMs), which in turn promote efferocytosis. This interaction ultimately facilitates the resolution of inflammation.

Lysophosphatidic acid (LPA), lysophosphatidylcholine (LPC), and sphingosine-1-phosphate (S1P) function as “find me” signals, facilitating the migration of macrophages toward apoptotic cells (Peter et al., 2008; Park and Kim, 2017; Geraldo et al., 2021). LPC is synthesized by apoptotic cells via the PLA2 enzyme and interacts with the G2A receptor on macrophage surfaces, thereby enhancing their chemotactic response (Gheibi Hayat et al., 2019). S1P attracts macrophages by binding to its receptor S1PR1 (Syed et al., 2019). Furthermore, S1P can activate downstream signaling pathways of S1PR1 in macrophages, resulting in the production of erythropoietin (EPO). Through paracrine/autocrine mechanisms, EPO signaling subsequently activates and upregulates efferocytosis-related molecules via peroxisome proliferator-activated receptor gamma (PPARg) activation, including Mer tyrosine kinase (MerTK) and growth arrest-specific 6 (Gas6), among others, thus priming the cells for efficient efferocytosis (Poon and Ravichandran, 2024). Phosphatidylserine (PS), a well-established “eat me” signal existing on the surface of apoptotic cells, is crucial for the recognition and phagocytosis of apoptotic cells by phagocytes, engaging with receptors on phagocytes and initiating the engulfment and internalization of apoptotic cells (Kay and Grinstein, 2013). Additionally, the activation of phospholipase A2 (PLA2) hydrolyzes esterified AA on the inner surface of cell membrane into free form (Wang et al., 2021). Then, arachidonic acid (AA) and its metabolites, such as prostaglandins and leukotrienes (Ortiz-Placín et al., 2023; Zhang et al., 2023), modulate the inflammatory response during efferocytosis. Free arachidonic acid (AA) produces prostaglandin E2(PGE2) under the action of cyclooxygenase (COX) (Burkett et al., 2023). It can attenuate inflammatory responses and promote the polarization of macrophages to the M2 phenotype (Wang et al., 2023a) (Figure 1).

### 2.2 Fatty acid oxidation (FAO) provides energy support for efferocytosis and influences macrophage polarization

The process of efferocytosis requires significant lipid remodeling (Teplova et al., 2013). Phagocytes are attracted to apoptotic cells, migrate towards them, and upon identifying the “eat me” signals on their surfaces, undergo cytoskeletal changes to internalize and engulf these cells. This process demands substantial energy. While glucose metabolism is a traditional energy source, fatty acid oxidation (FAO) is also essential to fulfill these energy requirements. Research indicates that during the engulfment of apoptotic cells by macrophages, there is a notable increase in mitochondrial fatty acid β-oxidation, which generates ATP to support phagosome formation and cytoskeletal restructuring. Consequently, FAO is crucial for providing the energy necessary for efferocytosis, ensuring the effective clearance of apoptotic cells (Chi et al., 2020). The enzyme carnitine palmitoyltransferase 1a (CPT1a) is integral to FAO. CPT1a-mediated FAO serves as a critical energy source for sustained efferocytosis in macrophages (Wang et al., 2024). Inhibition of the FAO pathway, either through the use of the CPT1a inhibitor Etomoxir or genetic knockout of CPT1a, causes a shift in macrophage energy metabolism towards glycolysis, resulting in mitochondrial dysfunction and a significant reduction in their ability to engulf apoptotic cells (Wang et al., 2024). In summary, a large amount of ATP produced by fatty acid oxidation provides energy for the formation of phagocytes and cytoskeleton remodeling during burial. At the same time, fatty acid oxidation can also maintain the steady state of mitochondria through CPT1a-IL-10 axis to ensure the continuous supply of energy (Figure 1).

Additionally, metabolic reprogramming during efferocytosis affects macrophage phenotypes: pro-repair (M2) macrophages rely on FAO, whereas pro-inflammatory (M1) macrophages depend on glycolysis (Huang et al., 2014). Studies suggest that in macrophages stimulated by IL-4, enhanced FAO and the associated spare respiratory capacity (SRC) are dependent on the lysosomal enzyme LAL, which mediates intracellular lipid degradation (Namgaladze and Brüne, 2016). The production of fatty acids to fuel FAO is consistent with the importance of FAO in M2 activation. The expression of the lysosomal enzyme LAL is more pronounced in M2 than in M1 macrophages and is essential for the response and activation of IL-4-induced M2 macrophages (Huang et al., 2014) (Figure 1).

### 2.3 Phagocytes maintain efferocytosis by preserving cholesterol homeostasis

Macrophages utilize various pathways to process and digest cargo from apoptotic cells, thereby protecting themselves from the stress associated with apoptotic cell load and ensuring the continuous process of efferocytosis, such as through metabolism or extrusion. Cholesterol, a major component of mammalian cell membranes, plays critical structural and functional roles. Excessive cholesterol accumulation is toxic to cells and forms the molecular basis of numerous diseases; thus, cellular cholesterol is tightly regulated to maintain homeostasis in the body (Song et al., 2021). In phagocytes, cholesterol is maintained in a relatively stable state to ensure the smooth progression of efferocytosis. Research has indicated that cholesterol efflux is essential for the recognition of phosphatidylserine (PS) exposure. In phagocytes, acyl-CoA:cholesterol acyltransferase (ACAT) can esterify free cholesterol derived from apoptotic cells into cholesterol esters, which can then be stored or released by ATP-binding cassette transporter A1 (ABCA1) (Fond et al., 2015). Phagocytes export cholesterol via ABCA1/ABCG1 (ATP binding cassette subfamily G member 1) transporters to apolipoprotein A-I (apoA-I), maintaining cell membrane fluidity and promoting effective localization of PS receptors (Yu and Tang, 2022). Additionally, oxysterols (such as 27-hydroxycholesterol) released by apoptotic cells can activate liver X receptors (LXR), inducing the expression of anti-inflammatory factors (such as IL-10) and phagocytic receptors (such as MerTK). Some studies suggest that after apoptotic cells are degraded in phagolysosomes, cholesterol is transported into the efferocytosis process. Once delivered, these cholesterols activate nuclear cholesterol receptors (such as liver X receptor-α [LXRα] and peroxisome proliferator-activated receptors [PPAR]γ and PPARδ). Upon activation of these receptors, anti-inflammatory cytokines such as IL-10 and TGFβ are produced, promoting the resolution of inflammation (Vafadar et al., 2024). Under pathological conditions, cholesterol homeostasis is disrupted, and the efferocytosis function of macrophages is similarly affected. For example, in atherosclerosis, when macrophage uptake of modified lipoproteins exceeds cholesterol efflux, cholesterol accumulates, leading to the formation of foam-like macrophages (Ye et al., 2019) (Figure 1).

### 2.4 The lipid sensor Trem2 on the surface of phagocytes influences efferocytosis by regulating lipid metabolism

Triggering receptor expressed on myeloid cells 2 (Trem2) functions as a cell surface lipid sensor that plays a regulatory role in microglial activity (Damisah et al., 2020). Research has indicated that Trem2 is also significant in macrophages, contributing to lipid metabolism and the maintenance of tissue homeostasis. Lipid-associated macrophages (LAMs) are a type of phagocytic cell adept at managing lipids, with the lipid receptor Trem2 being a primary driver of the adipose tissue macrophage response during obesity. LAMs can uptake lipids in a TREM2-dependent manner, and their role in regulating lysosomal lipid metabolism is conserved across both humans and mice. Furthermore, the expression of Trem2 on macrophages can mitigate the progression of metabolic disorders by facilitating the formation of crown-like structures around lipid-rich and dying adipocytes (Jaitin et al., 2019). The accumulation of extracellular lipids and inflammation are prevalent characteristics of obesity, progressive neurodegeneration, and atherosclerosis. The Trem2 signaling pathway modules may represent a conserved, general macrophage response employed to detect extracellular pathogenic lipids in various tissues. Consequently, Trem2 can function as a pattern recognition receptor indicating the disruption of tissue homeostasis (Jaitin et al., 2019). Some studies have suggested that Trem2 acts as a regulatory factor promoting tissue differentiation in foamy macrophages. In Trem2-deficient foamy macrophages, the cholesterol biosynthesis pathway cannot be downregulated, leading to impaired lipid uptake, increased mortality of foamy macrophages, inhibited clearance of apoptotic cells, and ultimately, the development of atherosclerosis (Patterson et al., 2023) (Figure 1).

TREM2 mutation has been identified as a risk factor for Alzheimer’s disease (AD) and other neurodegenerative diseases (NDD). Because TREM2 encodes a receptor that is only expressed on immune cells, the identification of these mutations finally shows that immune response can play an active role in the pathogenesis of NDD. These TREM2 variants also give the highest risk of Alzheimer’s disease among any risk factors found in recent 20 years (Jay et al., 2017). In nonalcoholic steatohepatitis (NASH, also known as metabolic dysfunction-related steatohepatitis, MASH), macrophages expressing Trem2 play a key role in promoting its development into hepatocellular carcinoma (HCC). Trem2 is the central regulator of NASH-driven immunosuppression niche of HCC (Wang et al., 2025). Atherosclerosis is a chronic disease of the vascular wall driven by lipid accumulation and inflammation in the intimal layer of arteries. Studies have shown that hematopoietic or global TREM2 deficiency increased, whereas TREM2 agonism decreased, necrotic core formation in early atherosclerosis. TREM2 is essential for the efferocytosis capacities of macrophages and to the survival of lipid-laden macrophages, indicating a crucial role of TREM2 in maintaining the balance between foam cell death and clearance of dead cells in atherosclerotic lesions, thereby controlling plaque necrosis (Piollet et al., 2024).

TREM2 receptor consists of three different domains, which can bind different ligands under various physiological or pathological conditions. Under normal circumstances, trem2 receptor interacts with lipoprotein and apolipoprotein E (APOE). It can combine with lipopolysaccharide (LPS) during tissue injury and pathogen invasion. Under the condition of AD, it directly binds to pathological β -amyloid (Aβ) oligomer (Que et al., 2025). The researchers found that TREM2 is a sensor for a variety of acidic and zwitterionic lipids, which may or may not contain phosphate moiety. The membrane containing these lipids strongly interacts with Aβ, which can promote the transformation of fibrous Aβ from Aβ peptide (Wang et al., 2015). TREM2 plays a double-edged role in microglia. First of all, TREM2 and its ligands DAP12 and ApoE are the main ways to transform microglia from a steady state to a state related to nervous system diseases. DAP12 is one of the important ligands of TREM2, and it is the main switch to change microglia from steady state to disease-related state. It increases the production of Aβ plaque and the diffusion of Tau protein, accelerates the pathological and behavioral defects of the brain, and ultimately increases the risk of many neurodegenerative diseases. Secondly, TREM2 can restore microglia homeostasis through TREM2-APOE-mediated pathway, and realize the synergistic effect of TREM2 and its ligand APOE by regulating the main transcription factors homologous to microglia (including TGF-β), thus increasing TREM2-mediated phagocytosis (Wei et al., 2024). Functional blockade of TREM2 exacerbates inflammation and demyelination while TREM2 deficiency diminishes the ability to maintain neural homeostasis, and leads to uncontrolled increases in neuroinflammatory responses through increased lysosomal activity. Prolonged inflammation can trigger a positive feedback loop, exacerbating symptoms of demyelinating diseases such as cognitive and motor impairments (Gao et al., 2023).

### 2.5 Efferocytosis can alleviate inflammatory responses through interactions with specialized pro-resolving mediators (SPMs)

The β-oxidation of fatty acids from apoptotic cells within the mitochondria leads to the production of NAD^+^ and complex III subunit 5, which in turn activate the transcription factor PBX1. This activation enhances the expression of the pro-resolving cytokine IL-10, thereby linking efferocytosis to the resolution of inflammation (Zhang et al., 2019). A key component in resolving inflammation is the conversion of polyunsaturated fatty acids into SPMs, such as lipoxins (LX), resolvins (RV), protectins (PT), and maresins. The synthesis and breakdown of these lipid mediators are essential for driving the resolution of inflammation (Pan et al., 2022). Efferocytosis contributes to the generation of SPMs through two primary mechanisms: the degradation of metabolic products from apoptotic cells and the enzymatic conversion of these precursors into mature lipid mediators, as well as by increasing the availability of SPM precursors like arachidonic acid (AA), eicosapentaenoic acid (EPA), and docosahexaenoic acid (DHA) (Doran et al., 2020). Additionally, the activation of the MerTK receptor through its interaction with apoptotic cells triggers the translocation of the 5-LOX enzyme (5-lipoxygenase) from the nucleus to the cytoplasm. In the cytoplasm, it collaborates with 12/15-LOX to convert AA to lipoxin A4 (LXA4) and DHA to resolvin D1 (RvD1), both of which are effective in suppressing and resolving inflammation (Schilperoort et al., 2023b). The interaction between phagocytes and apoptotic cells (AC) enhances the production of specific SPMs while reducing pro-inflammatory leukotrienes, thereby promoting the resolution of inflammation (Fredman and Tabas, 2017). Efferocytosis also upregulates the expression of 12/15-LOX, leading to increased SPM production (Schif-Zuck et al., 2011). Moreover, LXA4 and RvD1 further enhance efferocytosis, suggesting a positive feedback loop exists between efferocytosis and SPM production (Dalli and Serhan, 2016) (Figure 1).

## 3 Glucose metabolism and efferocytosis

### 3.1 SLC7A11 is a brake for DC efferocytosis

Dendritic cells (DCs) represent a diverse group of phagocytes present in nearly all tissues. These cells express a variety of phagocytic and pathogen recognition receptors, contributing to tissue homeostasis by modulating both innate and adaptive immune responses (Cabeza-Cabrerizo et al., 2021). While the uptake of apoptotic cells by DCs has been acknowledged for some time, research has predominantly concentrated on its implications for antigen presentation and adaptive immunity (Albert et al., 1998; Gallucci et al., 1999; Guermonprez and Amigorena, 2005; Blander and Medzhitov, 2006). In contrast to macrophages, the molecular mechanisms governing efferocytosis in DCs and its significance in inflammation remain less understood. The SLC family is the second largest gene family in the human genome after the G protein-coupled receptors, and it mediates the transport of metabolites and solutes across the cell membrane. SLC is associated with more than 100 human diseases and is also related to the efferocytosis of other phagocytic cells (Maschalidi et al., 2022). By detecting the exocytosis of BMDCs after siRNA-mediated knockdown of the SLC7 family, the results showed that interfering with the expression of SLC7A11 would enhance the efferocytosis of DCs. The subsequent experimental results also indicated that SLC7A11 is the molecular brake for the efferocytosis of DCs.

**FIGURE 2:**
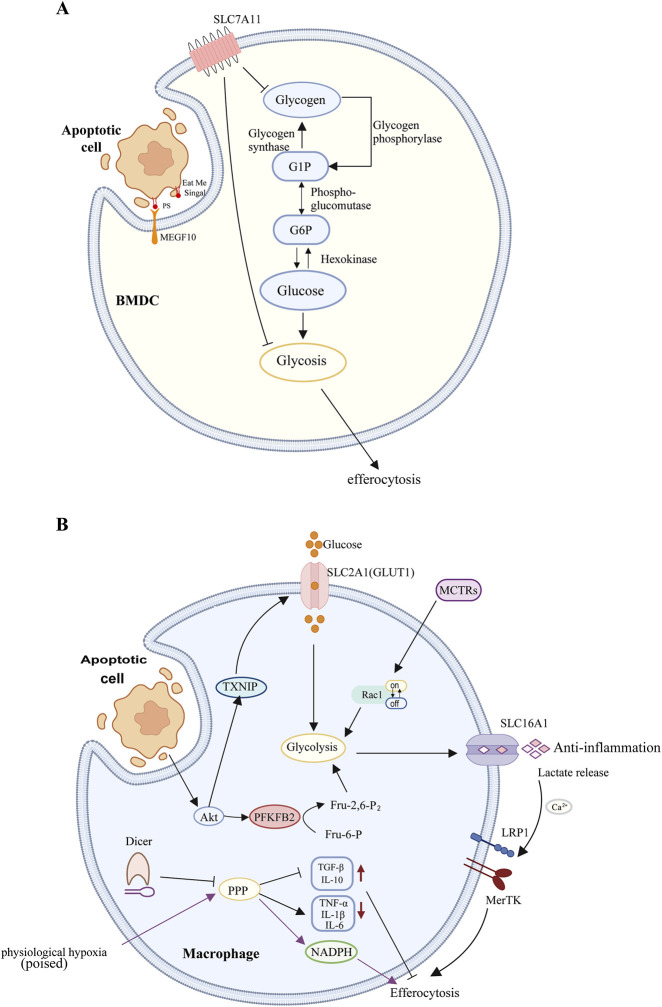
**(A)** Macrophages: During aerobic glycolysis, the uptake and utilization of glucose, mediated by SLC2A1, facilitate actin polymerization during phagocytosis. Concurrently, lactate release mediated by SLC16A1 promotes the polarization of immature macrophages and the release of anti-inflammatory factors, thereby aiding in the clearance of apoptotic cells. The phagocytosis of apoptotic cells by macrophages stimulates Akt dephosphorylation, leading to the phosphorylation and subsequent degradation of TXNIP. This process releases the glucose transporter GLUT1, increasing glucose uptake and thereby enhancing glycolysis. Akt-mediated phosphorylation of PFKFB2 catalyzes the production of Fru-2,6-P2, which activates phosphofructokinase-1 (PFK-1), further promoting glycolysis. Lactate produced during glycolysis can increase the surface expression of phagocytic receptors MerTK and LRP1 in a calcium-dependent manner, thereby facilitating the sustained uptake of apoptotic cells. MCTRs can promote macrophage phagocytosis by regulating Rac1 activity and upregulating glycolysis. Dicer inhibits the phagocytic function of macrophages and their inflammatory response during the clearance of apoptotic cells by suppressing the expression of PPP-related genes. Under chronic physiological hypoxia, macrophages can effectively shunt glucose into the PPP, and the NADPH generated by the PPP can protect macrophages from excessive lysosomal acidification and oxidative stress, thereby enhancing their efferocytosis and improving cellular adaptability to maintain critical homeostatic functions. **(B)** Dendritic Cells: SLC7A11 inhibits the phagocytosis of apoptotic cells by dendritic cells by suppressing the conversion of glycogen stored in DCs into glucose and the subsequent process of aerobic glycolysis.

SLC7A11 is a component of the cystine-glutamate antiporter system xc−, which regulates the exchange of intracellular glutamate and extracellular cystine. Additionally, SLC7A11 is implicated in neurological disorders (Merckx et al., 2017), viral infections (Kandasamy et al., 2016; Rabinowitz et al., 2021), and cancer (Koppula et al., 2021). The authors compared glycogen levels in the control group, SLC7A11-KO dendritic cells, and wild-type dendritic cells before and after erastin treatment. The results demonstrated that both the SLC7A11-KO group and the erastin-treated group exhibited reduced intracellular glycogen, and the introduction of a glycogen phosphorylase inhibitor reversed the effect of erastin. These findings suggest that glycogenolysis indeed contributes to increased efferocytosis. To determine whether aerobic glycolysis is involved in the enhanced efferocytosis observed in SLC7A11-deficient DCs, several pharmacological inhibitors—2-DG and 3-BP—were employed to suppress aerobic glycolysis. Both inhibitors mitigated the efferocytosis-enhancing effect of erastin. In conclusion, the conversion of glycogen to glucose and the subsequent process of aerobic glycolysis promote efferocytosis in SLC7A11-deficient dendritic cells (Maschalidi et al., 2022) (Figure 2A). Studies have shown that some diseases, such as stroke, patients receiving insulin therapy, mental illness and neurodegenerative diseases, have impaired brain glucose metabolism, which leads to insufficient brain glucose supply. This is a part of the pathological mechanism of ischemic diseases and also a manifestation of GLUT1 deficiency, which may further damage microglia phagocytosis and neuroimmunity, thus aggravating the pathological process of the disease (Rana et al., 2024).

### 3.2 Glycolysis facilitates efferocytosis by modulating actin polymerization and the expression of anti-inflammatory genes in adjacent cells

Solute carrier (SLC) proteins, located in the plasma membrane, mitochondrial membrane, and other internal membranes, are responsible for the transmembrane transport of molecules such as glucose, nucleotides, and amino acids (Mueckler and Thorens, 2013; César-Razquin et al., 2015; Rives et al., 2017). The solute carrier membrane transporter SLC2A1, also referred to as the glucose transporter GLUT1, is the most ubiquitously distributed glucose transporter in mammalian cells and is evolutionarily conserved (Yao et al., 2023). Research has demonstrated that SLC2A1 is significantly upregulated early in efferocytosis in LR73 cells and macrophages, enhancing the phagocytic capacity to engulf apoptotic cells by increasing glucose uptake. The internalization of apoptotic cells necessitates extensive actin polymerization (Nagata, 2018), which is associated with aerobic glycolysis during cell migration (Hu et al., 2016). During aerobic glycolysis, glucose uptake and utilization mediated by SLC2A1 contribute to actin polymerization during efferocytosis. Subsequent RNA-seq analysis of phagocytes engaged in engulfment revealed significant upregulation of multiple glycolytic genes. SLC16a1 functions as a plasma membrane proton-linked monocarboxylate transporter for lactate and pyruvate (Sprowl-Tanio et al., 2016). Evidence suggests that knockdown of SLC16a1 diminishes efferocytosis *in vitro* and facilitates lactate release from phagocytes (Morioka et al., 2018). As a glycolysis byproduct, lactate can induce tumor-associated macrophages (TAMs) and naive macrophages to polarize into M2 macrophages (Colegio et al., 2014). Efferocytic phagocytes can enhance the clearance of apoptotic cells by releasing lactate via SLC16A1, thereby polarizing naive macrophages, which subsequently secrete anti-inflammatory factors such as TGFβ and IL-10. In summary, aerobic glycolysis induced during efferocytosis influences efferocytosis on two levels: regulating efferocytosis through actin polymerization (involving SLC2A1) and promoting efferocytosis by modulating the expression of anti-inflammatory genes in neighboring cells (involving SLC16A1) (Morioka et al., 2018) (Figure 2B).

### 3.3 PFKFB2-mediated glycolysis promotes macrophage lactate-driven sustained efferocytosis

Previous studies have identified an association between both pro-inflammatory and efferocytic macrophages and increased glucose uptake, as well as elevated transcription levels of SLC2A1 (Morioka et al., 2018). It has been demonstrated that thioredoxin-interacting protein (TXNIP) functions as an endocytic adaptor for GLUT1, facilitating the removal of GLUT1 from the cell surface and thereby negatively regulating glucose uptake (Wu et al., 2013). The phagocytic action activates Akt by phosphorylating it, which is essential for effective efferocytosis (Yancey et al., 2010; Nishi et al., 2019; Gerlach et al., 2021). The activated Akt phosphorylates TXNIP, leading to its degradation. This maintains the cell surface expression level of GLUT1 and increases glucose intake (Wu et al., 2013; Waldhart et al., 2017). PFK1, a key rate-limiting enzyme in the aerobic oxidation of carbohydrates, catalyzes the phosphorylation of fructose-6-phosphate at the first carbon to yield fructose-1,6-bisphosphate (Icard et al., 2021). Its kinase activity is activated by fructose-2,6-bisphosphate, synthesized by the PFKFB enzyme family. Within this family, PFKFB2 is notably upregulated during efferocytosis, including its activated Ser485-phosphorylated form. There is evidence suggesting that various stimulating factors such as adrenaline, insulin, and hypoxia can cause the phosphorylation of PFKFB2 protein in the myocardium, thereby increasing the concentration of 2,6-diphosphofructose and promoting the glycolysis process. These effects are mediated by protein kinases (including PKA, Akt, and AMPK) that phosphorylate PFKFB2 at its C-terminal domain (Lee et al., 2020). Efferocytosis-induced Akt activation promotes two sequential steps in glycolysis: phosphorylation of TXNIP enhances GLUT1-mediated glucose uptake, and phosphorylation of PFKFB2 further augments glycolysis. These mechanisms are distinct from those observed during inflammation-induced glycolysis in macrophages. Consequently, efferocytosis promotes glycolysis through at least two mechanisms: the loss of TXNIP function to enhance glucose uptake and the potentiation of downstream glycolysis. Lactate produced during glycolysis can increase the cell-surface expression of the phagocytic receptors MerTK and LRP1 in a calcium-dependent manner, thereby promoting sustained uptake of apoptotic cells (Schilperoort et al., 2023a) (Figure 2B).

Another member of this family, PFKFB3, is frequently over-expressed in many human tumors, including lung, breast, colon, pancreas, ovary, pancreas and thyroid tumors, but under-expressed in normal tissues, so targeting PFKFB3 to treat cancer has become an attractive strategy (Wang et al., 2020). Infantile hemangioma (IH) is the most common tumor in infants, but the exact pathogenesis of IH is largely unknown. Studies have shown that by using PFK15 as an inhibitor, inhibiting PFKFB3 can inhibit PI3K-Akt signaling pathway downstream and induce apoptosis and death. More importantly, using PFK15 or shPFKFB3 to inhibit PFKFB3 can significantly reduce tumor growth *in vivo*. PFKFB3 inhibition can inhibit the angiogenesis of IH and induce apoptosis, which can effectively improve cancer (Yang et al., 2023). In addition, PFKFB3 plays the same important but different functions in macrophages and dendritic cells. Studies have shown that cytoplasmic virus recognition leads to the upregulation of glycolysis of macrophages through secondary interferon signal transduction. PFKFB3-driven glycolysis can phagocytize and remove virus-infected cells through metabolic support, and selectively promote the exogenous antiviral ability of macrophages (Jiang et al., 2016). Inflammatory corpuscles are described as cytoplasmic regulators of inflammation, and nucleotide binding domain-like receptor Family Pyrin domain containing 3 (NLRP 3) is the most important inflammatory corpuscles. Studies have shown that the production and release of interleukin-1β (IL-1β) depends on the activation of inflammatory corpuscles of NLRP3, and its activation and subsequent release of IL-1β play a key role in regulating glycolysis through PFKFB3. The NLRP3 inflammatory corpuscle -IL-1β-PFKFB3 axis is a key regulator of glycolysis of macrophages, and targeting this axis may help to alleviate inflammation and restore the metabolic balance of macrophages (Finucane et al., 2019). In dendritic cells, the use of PFKFB3 inhibitor PFK15 will inhibit the glycolysis and function of DCs, thus affecting the effect of cancer immunotherapy. However, by delivering fructose −1,6- diphosphate (F16bp, downstream product of PFKFB3), the glycolytic ability and function of DCs can be “saved”, the anti-tumor response of activating cytotoxic T cells (Tc) can be enhanced, and the survival of mice can be improved (Inamdar et al., 2023).

### 3.4 Efferocytosis-derived MCTRs metabolically promote sustained macrophage efferocytosis via Rac1-mediated glycolytic activation

The continuous engulfment of multiple apoptotic cells by phagocytes is crucial for maintaining organ function, resolving acute inflammation, and facilitating tissue repair. This process necessitates coordinated cellular responses to ensure the recognition and clearance of target cells, effective metabolic processing of the load, and adequate energy availability to sustain the process (Tabas, 2010). Recent studies have demonstrated that maresin conjugates in tissue regeneration (MCTRs) can enhance sustained macrophage efferocytosis during tissue regeneration (Xiao et al., 2021). This enhancement occurs through the initiation of apoptotic cell uptake by macrophages and an increase in the proportion of macrophages engaged in efferocytosis (Koenis et al., 2024). Phosphoproteomic analyses of MCTR-treated macrophages have identified several glycolytic enzymes with differential phosphorylation. To explore the impact of MCTRs on glycolytic metabolism, the authors examined the steady-state levels of various glycolytic and TCA cycle metabolites in human macrophages treated with MCTRs in the absence of apoptotic cells. The findings indicated that MCTR treatment elevated the levels of glucose-6-phosphate, fructose-1,6-bisphosphate, 3-phospho-D-glycerate, and phosphoenolpyruvate, suggesting enhanced glycolysis. However, this MCTR-induced increase in glucose uptake was nullified in macrophages treated with a Rac1 inhibitor. Rac1 has been previously shown to regulate glycolytic flux by releasing aldolase (a key glycolytic enzyme) from the cytoskeleton, thereby facilitating the actin rearrangement necessary for apoptotic cell uptake (Nakaya et al., 2008) and activating glycolytic metabolism (Hu et al., 2016). Seahorse flux analysis of macrophages pre-treated with Rac1 or aldolase inhibitors revealed that the MCTR-mediated increase in macrophage glycolytic capacity requires both Rac1 and aldolase activity. These findings demonstrate that MCTRs promote macrophage efferocytosis by modulating Rac1 activity and upregulating glycolysis in macrophages (Koenis et al., 2024) (Figure 2B).

### 3.5 Macrophage dicer promotes the clearance of tolerogenic apoptotic cells and immune tolerance by inhibiting the pentose phosphate pathway activity

Dicer, a member of the RNase III family, is recognized for its function as a ribonuclease in microRNA biogenesis (Ha and Kim, 2014). While recent studies have elucidated the link between microRNA and apoptotic cell (AC) clearance, the specific interaction between Dicer and efferocytosis remains unexplored (Tajbakhsh et al., 2019). Research indicates that a deficiency in Dicer results in a marked decrease in the phagocytosis rate of ACs by macrophages. Furthermore, compared to wild-type (WT) macrophages, Dicer-deficient macrophages exhibit increased expression of TNF-α, IL-1β, and IL-6, alongside decreased levels of TGF-β and IL-10, suggesting an enhanced inflammatory response during AC clearance. Gene set enrichment analysis has demonstrated a significant enrichment of pentose phosphate pathway (PPP) gene signatures in Dicer-knockout (KO) macrophages, a phenomenon not observed in WT cells. Additionally, the expression of seven PPP-related genes and the levels of their products—NADPH and glutathione—(Nagy and Haschemi, 2015) were significantly elevated during efferocytosis compared to control groups. Notably, even in the absence of AC stimulation, Dicer deficiency alone augments the expression of PPP-related genes and the levels of NADPH and glutathione. Treatment with PPP antagonists, dehydroepiandrosterone (DHEA) and 6-aminonicotinamide (6-AN), restored the phagocytic capacity of Dicer-KO macrophages to WT levels and mitigated the inflammatory response during efferocytosis (Mao et al., 2021) (Figure 2B).

### 3.6 Efferocytosis in hypoxic environments

Tissue-resident macrophages (TRMs) persist within tissues throughout the organism’s lifespan (Okabe and Medzhitov, 2016; Guilliams et al., 2020; Guilliams and Svedberg, 2021). As a vital component of the immune microenvironment, TRMs constitute a self-renewing and long-lived cell population that plays a pivotal role in maintaining homeostasis, facilitating tissue repair post-injury, mitigating inflammation, and even modulating cancer progression (Cao et al., 2024). Notably, nearly every organ under steady-state conditions harbors distinct types of TRMs, which are adept at sensitively detecting environmental changes and swiftly adapting to maintain tissue health (Lazarov et al., 2023; Mass et al., 2023). They accomplish the clearance of tissue-specific debris or by-products—including dying or dead cells and tissue-specific cellular secretions—through various mechanisms, including phagocytosis and efferocytosis (Nicolás-Ávila et al., 2020; Brestoff et al., 2021; Schilperoort et al., 2023b). Chronic physiological hypoxia can induce macrophages to exhibit two distinct yet complementary states. The first, termed the “primed” state, involves the transcription and translation of metabolic programs in AC-naive macrophages that persist during efferocytosis. The second, the “poised” state, involves the transcription (but not translation) of phagocytic functional programs in AC-naive macrophages during efferocytosis. Both states are essential for enhancing sustained efferocytosis (Wang et al., 2023b). Network analysis of differentially regulated metabolic programs in induced macrophages revealed significant differences in glucose uptake in macrophages within hypoxic environments; macrophages under chronic hypoxic conditions engage in less aerobic glycolysis compared to those cultured under normoxic conditions. Untargeted metabolomics analysis demonstrated a significant upregulation of the atypical pentose phosphate pathway (PPP), a reduction in tricarboxylic acid (TCA) cycle intermediates, and a notable accumulation of PPP intermediates, indicating that macrophages utilize glucose in a non-traditional manner. Macrophages exposed to prolonged hypoxia seldom use glucose for energy production via glycolysis. Instead, glucose is efficiently redirected to the PPP pathway to provide ribose-5-phosphate for new nucleotide synthesis and generate NADPH for maintaining redox balance. The data suggest that NADPH production mediated by the atypical PPP is crucial for the rapid maturation of phagolysosomes, protecting macrophages from excessive lysosomal acidification and oxidative stress, thereby enhancing macrophage efferocytosis and improving cellular adaptability to ensure the proper execution of critical homeostatic functions (Wang et al., 2023b) (Figure 2B).

Literature shows that hypoxia significantly increases the phosphorylation of p38-MAPK. At the same time, the inhibition of p38 reversed the effect of hypoxia on phagocytosis, indicating that p38 plays a role in the hypoxia regulation of phagocytosis. Hypoxia also significantly increased the expression of hypoxia-inducible factor -1α(HIF-1α) in macrophages, which was reversed after p38 inhibition, indicating that there was a relationship between p38 activation and HIF-1α expression. The knock-down of HIF-1α small interfering RNA reversed the effect of hypoxia on phagocytosis, and compared with the control group, the overexpression of HIF-1α led to an amazing increase in phagocytosis. Hypoxia enhances phagocytosis of macrophages in a HIF-1α-dependent manner, and surface HIF-1α plays an important role in host defense (Anand et al., 2007). In addition, it is found that acute hypoxia can inhibit the proliferation and phagocytosis of macrophages, and at the same time, the changes of transcriptome reprogramming and metabolic remodeling occur. Firstly, the pro-inflammatory response of macrophages is activated during acute hypoxia, accompanied by enhanced anti-inflammatory regulation mechanism. Secondly, besides the increase of glycolysis, the key intermediates in pentose phosphate pathway also increased significantly. If the sugars 1,6- diphosphate, 6- phosphate and 5- phosphate ribose, it shows that pentose phosphate pathway is an important compensatory metabolic regulation of macrophages to acute hypoxia. This study shows that acute hypoxia can change the transcriptome and metabolomics in specific inflammatory reactions and metabolic pathways, so as to promote the adaptation of macrophages under hypoxic conditions (Sun et al., 2025).

## 4 Amino acid metabolism and efferocytosis

### 4.1 Polyamines produced by metabolism of arginine derived from apoptotic cells enhance the continuous phagocytic capacity of macrophages

Macrophages utilize two distinct metabolic pathways for arginine. In inflammatory macrophages, arginine is catalyzed by inducible nitric oxide synthase (iNOS) to produce nitric oxide (NO). Conversely, in pro-resolving mouse macrophages, arginine is converted by arginase 1 (Arg1) into ornithine, a precursor of polyamines (Rath et al., 2014). Ornithine may be metabolized into citrulline via ornithine transcarbamylase (OTC) as part of the urea cycle or converted into polyamines through ornithine decarboxylase (ODC; gene name Odc1) (Kierszenbaum et al., 1987).

**FIGURE 3:**
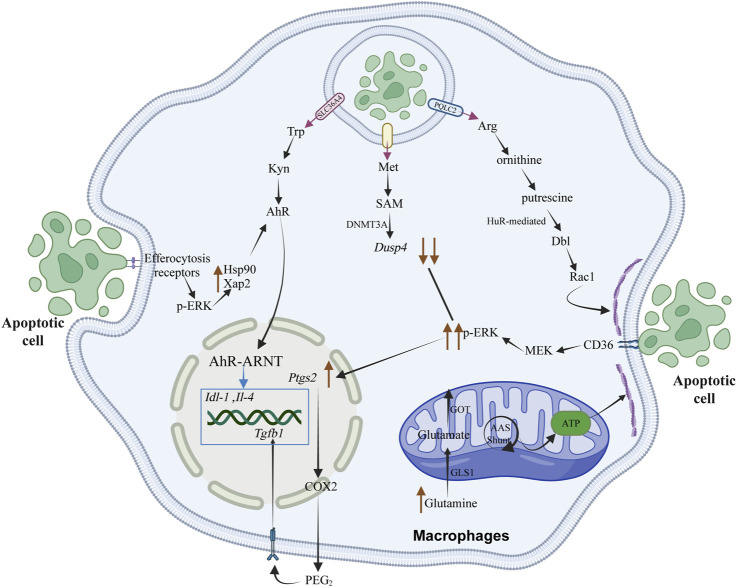
The metabolic products of amino acids, resulting from the degradation of apoptotic cells by macrophages, play a crucial role in regulating the resolution of inflammation and the efficiency of subsequent phagocytosis. This regulation occurs through the modulation of signal transduction processes, energy metabolism, and the transcription of anti-inflammatory and pro-repair genes within macrophages. Rac1 is instrumental in cytoskeletal remodeling by affecting the stability of Dbl mRNA, which encodes a GTP exchange factor. This factor activates the Rac1 protein, thereby stimulating cytoskeletal rearrangement in macrophages to enhance their efficiency in continuous phagocytosis. The tryptophan metabolite kynurenine (Kyn) can bind to the downstream aryl hydrocarbon receptor (AhR), influencing signal transduction processes in macrophages and promoting the expression of anti-inflammatory and pro-repair genes, such as Il-4 and Tgfb1, thereby facilitating tissue repair. The methionine metabolite S-adenosylmethionine (SAM) undergoes epigenetic modification catalyzed by the methyltransferase DNMT3A, activating the downstream PGE2-TGFβ1 pathway and regulating the expression of anti-inflammatory and pro-repair genes. Elevated intracellular glutamine concentrations in macrophages are converted through a non-canonical transaminase pathway that integrates oxidative stress buffering with ATP production, thus meeting the high-energy demands of actin dynamics and cytoskeletal rearrangement.

Following phagocytosis in pro-resolving macrophages, these cells acquire unique programming capabilities. During the degradation of apoptotic cells, arginine derived from this process is metabolized by arginase 1 and ornithine decarboxylase to produce the polyamine putrescine, which functions as a regulatory factor affecting Rac1, thereby influencing cytoskeletal rearrangement and the uptake of a subsequent apoptotic cell. When macrophages are co-incubated with IL-4 and apoptotic cells, measurements of the polyamines—putrescine, spermine, and spermidine—reveal a significant increase in putrescine, while spermine and spermidine levels remain unchanged. Furthermore, silencing Arg1 or Odc1 prevents the increase of putrescine in AC + macrophages but does not significantly affect spermidine or spermine levels in these cells. This confirms the presence of the Arg1-ODC-putrescine signaling pathway (Yurdagul et al., 2020) (Figure 3).

### 4.2 Non-canonical glutamine metabolic pathways enhance macrophage phagocytic efficiency by influencing cellular detoxification and energy metabolism

In macrophages, glutamine converts to glutamate by glutaminase 1 (Gls1) (Benhmammouch and Yvan-Charvet, 2023). Glutamine undergoes conversion via non-classical transaminase pathways, coupling oxidative stress buffering with ATP production for actin dynamics and cytoskeletal reorganization. Glutamine can be converted by glutamate dehydrogenase (GLUD1), serve as a GSH synthesis precursor through non-classical pathways, or transform via transaminases. Classical glutamine catabolism supports α-ketoglutarate-mediated coordination of macrophage activation through metabolic and epigenetic reprogramming (Liu et al., 2017). However, studies have not observed disruptions in α-KG-dependent epigenetic activity, and α-ketoglutarate supplementation does not rescue phagocytic defects in Gls1-deficient macrophages (Merlin et al., 2021). Compared to Gls1 deficiency, GLUD1-deficient macrophages show increased phagocytic activity, suggesting reduced glutamate in Gls1-deficient macrophages affects the non-classical glutamine catabolic pathway. High-throughput analyses reveal that glutamine catabolism in Gls1-deficient macrophages primarily disrupts downstream non-classical pathways of GSH synthesis and transaminase function. GLUD1-deficient macrophages have higher OCR, maximal respiratory response, ATP production rate, and phagocytic activity compared to Gls1-deficient ones. Studies confirm that glutamine catabolism limits oxidative stress and supports mitochondrial OXPHOS and ATP generation in reparative macrophages.

The malate-aspartate shuttle, facilitated by transaminases, functions in the aspartate-arginine-succinate bypass system. This mechanism requires glutamate to generate NADH, which is used in mitochondrial electron transport for energy production and ATP synthesis. The malic enzyme (ME)-dependent malate-pyruvate cycling pathway generates NADPH, supporting the compensatory pathway for reduced glutathione (GSH) synthesis (DeBerardinis and Cheng, 2010). In Gls1-deficient macrophages, ME1 and ME2 overexpression restores NAD(P)H levels and rescues phagocytic activity. This shows how non-classical glutamine metabolic pathways influence phagocytosis through cellular detoxification and energy metabolism. Glutamine hydrolysis modulates phagocytic response via post-transcriptional mechanisms. During phagocytosis, actin cytoskeleton remodeling in macrophages requires energy for actin polymerization, depolymerization, and membrane dynamics essential for recognizing and internalizing apoptotic cells (Elliott and Ravichandran, 2016). Gls1-deficient macrophages show slight downregulation of Cdc42 mRNA, a small G protein involved in actin polymerization, suggesting glutamine catabolism supports F-actin remodeling energy demands. In the presence of AhR (aryl-hydrocarbon receptor) ligands, these proteins are crucial for AhR ligand-induced nuclear translocation and activation. By activating Rac1, they facilitate cytoskeletal reorganization and enhance macrophage phagocytic capacity. In murine models, IDO1 (indoleamine 2,3-dioxygenase 1) or AhR deficiency impairs macrophage phagocytic efficiency. During atherosclerosis regression, IDO1 knockdown in macrophages obstructs plaque stabilization responses. Increased IDO1 mRNA expression and sustained phagocytosis reinforce each other, creating a positive feedback loop. This pathway promotes resolution and enhances efferocytosis *in vivo*, as observed during human atherosclerosis regression (Sukka et al., 2024) (Figure 3).

### 4.3 The phagocytosis-driven tryptophan metabolic pathway in macrophages promotes tissue repair

In humans, tryptophan is an essential amino acid that functions as a precursor for the synthesis of several significant bioactive compounds and plays a vital role in protein synthesis. Its metabolism occurs through three primary pathways: the kynurenine pathway, the indole pathway, and the 5-hydroxytryptamine (serotonin) pathway. The kynurenine pathway is the principal route of tryptophan metabolism, accounting for the degradation of over 95% of tryptophan into various bioactive compounds. Tryptophan 2,3-dioxygenase (TDO), indoleamine 2,3-dioxygenase 1 (IDO1), and IDO2 are key rate-limiting enzymes in the kynurenine metabolic pathway (Xue et al., 2023).

Over the past two decades, the roles of IDO1 and AhR in immunobiology have garnered considerable attention. Following the phagocytosis of apoptotic cells by macrophages, elevated levels of tryptophan have been observed in both human and murine macrophages. This increase is dependent on the upregulation of the membrane-bound tryptophan transporter SLC36A4 and intracellular IDO1 in macrophages during the degradation of apoptotic cells in phagolysosomes, which also results in increased levels of the tryptophan metabolite kynurenine (Kyn). The effect of Kyn was investigated by adding it to macrophages treated with IDO1 inhibitors, IDO1 knockout, or SLC36A4 silencing, to assess its role in sustained phagocytosis and in response to post-phagocytic supernatant induction. After the initial batch of apoptotic cells was introduced, Kyn was supplemented to macrophages treated with Epacadostat (an IDO1 inhibitor), and it was found that Kyn could partially restore sustained phagocytic activity. Kyn also partially restored the expression of Tgfb1 and IL10 in IDO1-KO macrophages. Upregulation of IDO1 induces increased AhR levels via Kyn-dependent activation. Upon activation, AhR initiates two key signal transduction pathways. First, the expression of IL10, Tgfb1, and Ido1 mRNA requires Kyn and ERK1/2-mediated upregulation of AhR chaperones HSP90 and XAP2. AhR forms a complex with HSP90 and XAP2; in the absence of a ligand, HSP90 and XAP2 anchor AhR in the cytoplasm, preventing its degradation by another protein called P23 and maintaining AhR in a high-affinity ligand-binding state (Pappas et al., 2018). Therefore, in the presence of AhR ligands, these proteins are essential for subsequent ligand-induced nuclear translocation and activation of AhR. Second, activation of Rac1 promotes cytoskeletal rearrangement and enhances the sustained phagocytic capability of macrophages. In mouse models of induced phagocytosis, deficiency of IDO1 or AhR impairs macrophage phagocytic efficiency. During atherosclerosis regression, macrophage IDO1 knockdown obstructs responses that contribute to plaque-stabilizing characteristics. The authors found that increased expression of Ido1 mRNA and sustained phagocytosis reinforce each other, establishing a beneficial positive feedback loop. This pathway promotes the resolution of inflammation and enhances efferocytosis *in vivo*, for instance, during significant disease processes such as the regression of atherosclerosis in humans (Sukka et al., 2024) (Figure 3).

### 4.4 Macrophages utilize methionine derived from the degradation of apoptotic cells and DNMT3A to facilitate tissue repair

In the context of amino acid catabolism, methionine (Met) is catalyzed by methionine adenosyltransferase (MAT) into S-adenosylmethionine (SAM). As a methyl donor, SAM is involved in various methyltransferase reactions and is converted to S-adenosylhomocysteine (SAH) during these processes. Methionine can enhance the concentration of glutathione within cells, promote cellular redox regulation, and protect cells by binding with oxidative metabolites (Ling et al., 2023). CD36 is a member of class B scavenger receptor family that recognizes apoptosis signal PS (Tait and Smith, 1999), and the interaction between apoptotic cells and CD36 mediated by PS can be conducted through CD36-ERK1/2 pathway, which regulates the synthesis of PEG2, thus affecting the synthesis of TGF-β1 (Xiong et al., 2013). During phagocytosis, the synthesis of prostaglandin E2 (PGE2) and transforming growth factor beta 1 (TGFβ1) is a critical step in the process (Fadok et al., 1998). PGE2 is synthesized from arachidonic acid (AA), which is released from membrane phospholipids through the action of phospholipase A2 (PLA2). Arachidonic acid is subsequently oxygenated by cyclooxygenase (COX) to form prostaglandin H2 (PGH2), which is then converted to PGE2-by-PGE2 synthase (PGES). Cyclooxygenase-2 (COX-2) serves as a rate-limiting enzyme for PGE2 synthesis and is reported to be highly induced by inflammation-related mediators following injury (Xiao et al., 2004). The deleterious effects suppressed by COX-2-derived prostacyclins may contribute to the susceptibility to diseases such as hypertension, increased risk of thrombosis, and atherosclerosis.

In the clearance of apoptotic cells in atherosclerosis disease models, the downstream signaling pathways (i.e., PGE2 expression process) activated by the CD36-ERK1/2 signaling pathway are limited by negative feedback regulation mediated by DUSP4. During the degradation of apoptotic cells after phagocytosis, methionine derived from this process is converted into SAM by MAT (methionine adenosyl transferase) in macrophages. SAM serves as a substrate for DNMT3A (DNA methyltransferase 3A) in DNA methylation modification, and Met/SAM acts as a rate-limiting factor for certain DNA methylation events in macrophages. When the inhibitor PF-9366 is used to suppress MAT2A or SiMat2a, the addition of exogenous SAM can induce ptgs2 and Tgfb1. Methylation-induced inhibition of Dusp4 prolongs ERK phosphorylation events, thereby allowing the induction of downstream ptgs2 (encoding COX2) and activation of the downstream PGE2-TGFβ1 pathway. In atherosclerotic mouse models, knocking out or silencing the DNMT3A gene in macrophages prevents the methylation of Dusp4, thereby hindering plaque regression in mouse atherosclerosis. In contrast, when Dusp4 is methylated normally, its expression decreases, and phosphorylated ERK remains active longer to induce ptgs2 expression, which subsequently activates the PGE2-TGFβ1 signaling pathway to promote tissue repair. Not only is DNMT3A required for SAM-mediated induction of ptgs2, but DNMT3A is also involved in the p-CREB pathway when PGE2 induces Tgfb1. This indicates that the DNMT3A-PEG-2-TGFβ1 pathway associated with epigenetic modifications plays an important role in diseases related to efferocytosis (Ampomah et al., 2022) (Figure 3).

## 5 Conclusion and future prospects

Efferocytosis is a critical immune regulatory process linked with metabolic reprogramming. Through molecular pathways, phagocytes transform metabolites from apoptotic cells into regulatory signals, aiding inflammation resolution and tissue repair as they clear cellular debris. Despite advances, several questions remain: How can single-cell multi-omics technologies explore metabolic flux during efferocytosis stages at single-cell and sub-organelle levels, such as mitochondria and lysosomes? How do organelles like mitochondria, lysosomes, lipid droplets, and endoplasmic reticulum collaborate in processing metabolites from apoptotic cells? What are the consequences of disrupted interactions on clearance functions? What are the patterns and functional differences of metabolic reprogramming induced by macrophages in different tissue microenvironments, such as microglia or Kupffer cells, and by apoptotic cells of diverse origins, like neutrophils or tumor cells? How do metabolites from efferocytosis, such as acetyl-CoA, α-ketoglutarate, and SAM, function as substrates to modify phagocyte gene expression at the epigenetic level through histone acetylation, methylation, or succinylation? How phagocytosis stimulates glucose metabolism, and how glucose metabolism regulates phagocytosis, remains to be further explored. Is it feasible to develop pharmacological strategies targeting phagocyte energy metabolism?

In summary, studying metabolism in efferocytosis reveals principles for homeostasis maintenance, provides new perspective on immune regulation, and enables innovative metabolic intervention strategies for diseases associated with efferocytosis.
